# Abnormal optical anisotropy in correlated disorder KTa_1−x_Nb_x_O_3_:Cu with refractive index gradient

**DOI:** 10.1038/s41598-018-20756-9

**Published:** 2018-02-13

**Authors:** Xin Zhang, Shan He, Zhuan Zhao, Pengfei Wu, Xuping Wang, Hongliang Liu

**Affiliations:** 1 Institute of Modern Optics, Nankai University, Tianjin, 300071 China; 2 Tianjin Key Laboratory of Optoelectronic Sensor and Sensing Network Technology, Tianjin, 300071 China; 3 Advanced Materials Institute, Shandong Academy of Sciences, Jinan, 250014 China

## Abstract

In this report, an abnormal optical anisotropy in KTa_1−x_Nb_x_O_3_:Cu (Cu:KTN) crystals with refractive index gradient is presented. Contrary to general regulation in a cross-polarization setup, the transmitted intensity of both TE (horizontally polarized) and TM (vertically polarized) lasers aligned with the basic crystallographic directions can be modulated quasiperiodically. The mechanism is supposed to be based on the polarization induced by the temperature gradient and the refractive index gradient. Meanwhile, the correlated disorder property of the crystals in the range of the freezing temperature (*T*_f_) and the intermediate temperature (*T* ^*^) also plays an important role. With the results verified both theoretically and experimentally, we believe this work is not only beneficial for the development of the theory associated with the correlated disorder structures in relaxor ferroelectrics, but also significant for the exploitation of numerous optical functional devices.

## Introduction

Correlated disorder, a peculiarity of relaxor ferroelectrics, has received tremendous attention in the research of functional devices in recent years^1–4^. In particular, among the numerous studies, amazing giant piezoelectric effect and excellent electro-optic (EO) effect have been extensively reported, which is significantly essential to practical applications^5–8^. The superior performances therein were mainly attributed to the microstructures associated with the correlated disorder state in the vicinity of the dielectric peak temperature (*T*_m_) below *T* ^*^ and above *T*_f_, defined as the PNDs by J. Toulouse^9^. However, the relationship between the unique nanostructures in these relaxors and the enormous predominant features has not been clearly explicated and remains to be a quite popular topic.

The optical anisotropy of single crystals deeply influences the polarization performance, which is of vital importance for the applications in kinds of optical elements and systems. KTa_1−*x*_Nb_*x*_O_3_ (KTN) is the solid solution of KTaO_3_ and KNbO_3_ and can be in its cubic, tetragonal or orthogonal phase at room temperature depending on the value of *x*, making it highly adaptable for different kinds of device applications. With the temperature across *T*_*m*_, Nb ions displace off from their original center sites, which induces spontaneous polarization with the anisotropy of the physical properties in certain direction^9^. Recently, the research on KTN crystals suffers a great explosion because of their strong EO and thermo-optic responses, showing impressive potential in beam scanning, EO modulation and high-resolution imaging and microscopy^10–19^. Meanwhile, the optical anisotropy was mostly involved in these investigations. Generally speaking, for a normally incident beam, the optical anisotropy is in line with the basic crystallographic directions of single crystals, leading to unequal refractive indexes for the corresponding polarization components. Thus, we can modulate optical parameters in respect of polarization^20^. Nevertheless, in this report, we observe an abnormal optical anisotropy, which is inconsistent with common cases. Lasers with both TE and TM polarizations can still be modulated quasiperiodically, despite their alignment with the basic crystallographic directions. The mechanism of this novel phenomenon is supposed to lie in the conjunction of the polarization induced by the temperature gradient and the refractive index gradient. In addition, the correlated disorder structure of the crystals as well plays an important role. The content herein will enrich related theories and experiences of ferroelectric relaxors in association with the correlated disorder structures. Besides, the abnormal optical anisotropy, unlimited to basic crystallographic directions, will render more flexible designs and applications for KTN crystals and other relaxors in the exploitation of optical functional devices.

## Results and Discussions

### Determination of *T*_f_ and *T* ^*^

**Figure 1:**
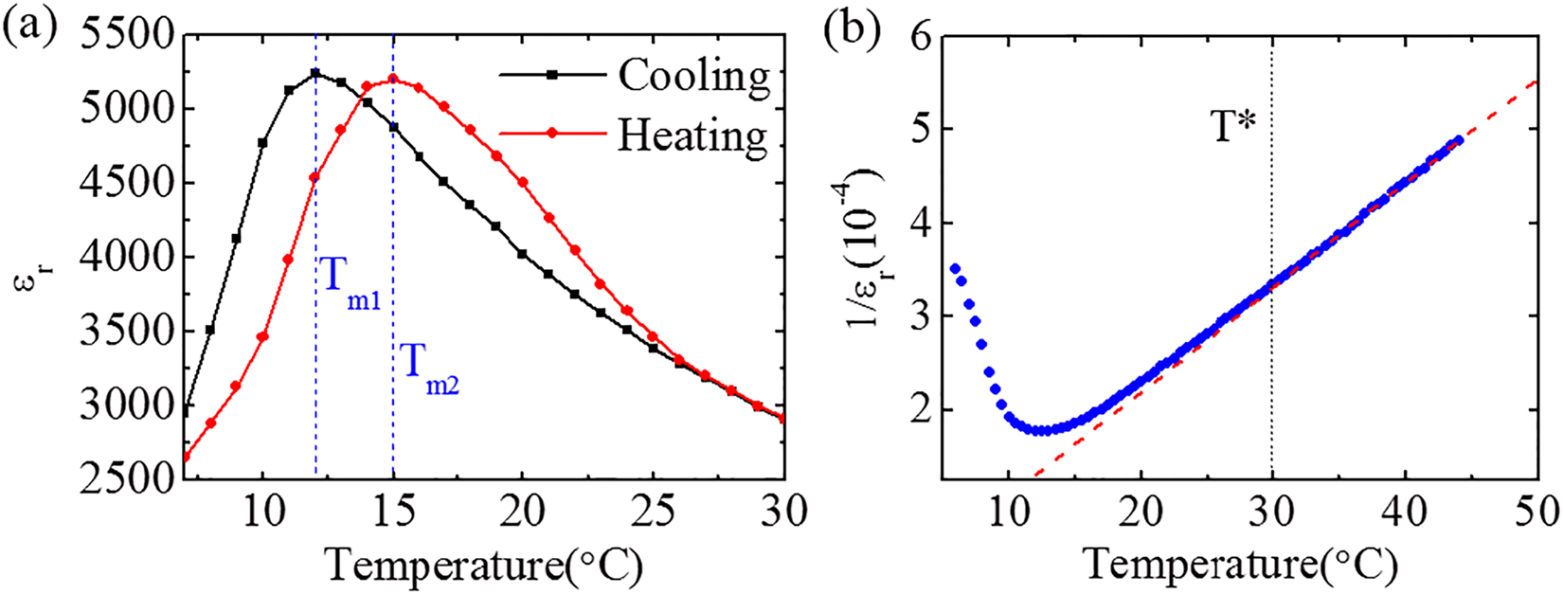
(**a**) The temperature-dependent relative dielectric constant for both slow cooling and heating (0.05 °C/s). (**b**) Curie-Weiss fitting of the 1/*ε*_r_-*T* curve indicating *T* ^*^ is around 30 °C.

Connected to an LCR (Inductance, Capacitance, Resistance) meter, the temperature-dependent relative dielectric constant (*ε*_r_) of the prepared crystal sample is measured (see Methods). As exhibited in Fig. 1(a), the temperature is controlled both decreasingly and increasingly between the range of 7 °C and 30 °C. The obvious thermal hysteresis declares a complex solid phase^21^, presaging that the operating temperature range exceeds *T*_f_. The dielectric peak temperatures are 12 °C and 15 °C for decreased and increased temperature circles, denoted as *T*_m1_ and *T*_m2_, respectively. Figure 1(b) demonstrates the relationship between the reciprocal of *ε*_r_ and the temperature from 7 °C to 45 °C, indicating that T^*^ is around 30 °C, obtained through the Curie-Weiss law^22^. Based on the above analysis, the temperature range of 7 °C and 30 °C is determined to locate between *T*_f_ and T^*^, where the correlated disorder state typically exists^9^.

### Investigation of the abnormal optical anisotropy

**Figure 2:**
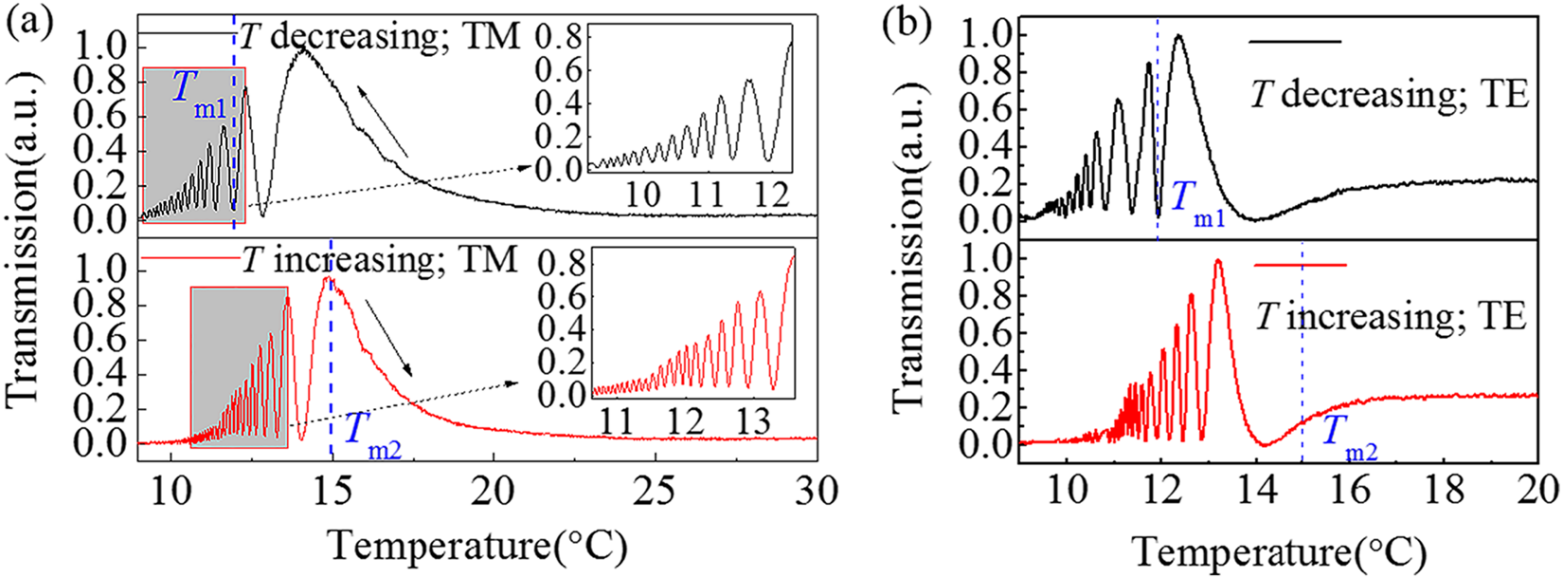
(**a**) The transmission for both decreased and increased temperature in the range of 9~30 °C with a 3 °C/min rate for TM-polarized lasers. (**b**) The transmission for both decreased and increased temperature in the 9~20 °C range with a 3 °C/min rate for lasers at TE polarization.

In this experiment, the sample is placed between a cross-polarization setup (see Methods) with its geometrical axes corresponding to the basic crystallographic directions. It should be noted here that the polarizer is along with either the *x* (vertical) or the *y* (horizontal) direction, instead of a 45° cross-polarization setup used in routine studies of optical anisotropy^23^. The signal of the transmitted light is collected by the detector and displayed in Fig. 2. As Fig. 2(a) shows, for TM-polarized lasers, the transmission suffers quasiperiodical oscillation when the temperature decreases across *T*_m1_ or increases across *T*_m2_, respectively. Besides, as Fig. 2(b) illuminated, TE-polarized lasers present a similar feature. Generally, the optical anisotropy should not arise for such a setup since the polarizer is located aligned with the basic crystallographic directions. However, extraordinary change of the transmission versus the temperature is observed in this work, which is named as the abnormal anisotropy.

### Depolarization of the incident lasers caused by the temperature gradient and the refractive index gradient

According to previous reports^24,25^, the orientation of the PNDs has a close relationship with the refractive index in the temperature range of [*T*_f_, T^*^]. Since the two orthogonally polarized beams are both responsive, there must be an orientation at a none-zero angle to either of the two crystallographic directions. In other words, a polarization field in the corresponding direction should exist to reorient the original randomly oriented PNDs. Thus, the depolarization of lasers appears in each crystallographic direction. As specified in the related report^26^, a pyroelectric field (***P***_Δ*T*_) induced by the temperature gradient was observed to form a polarization field in KTN crystals, effectively driving the PNDs to reorient coincidently. However, this can only partially explain the depolarization of TE-polarized lasers rather than lasers at TM polarization.

**Figure 3:**
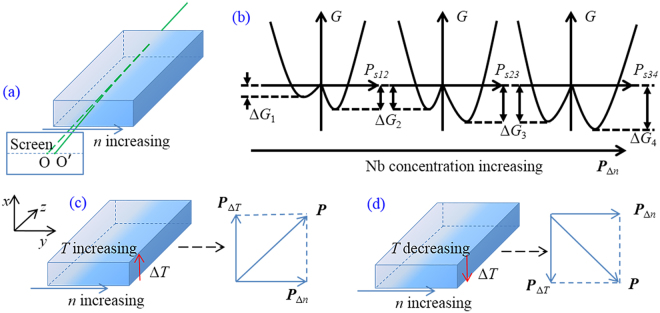
(**a**) A normally incident beam deviates from its original path, indicating a refractive index gradient **△***n* along the *y*-axis; (**b**) Free energy *G* for local regions and the polarization ***P***_**△***n*_ in association with Nb ions concentration. The temperature and refractive index gradient of the crystal and the corresponding polarization directions (in the *x-y* plane) for (**c**) increased and (**d**) decreased temperature.

In the measurement, a slight anomaly emerges that the transmitted beam deviates its original rectilinear path from *O* to *O*′, as shown in Fig. 3(a). Since we affirm the parallelism of the two faces is normal to the incident beam, a refractive index gradient, Δ*n*, should be involved in the *y-axis*^11,27^. Correspondingly, the refractive index gradient implies an incremental concentration of Nb ions for a positive Δ*n*^28,29^. According to the off-center theory^30–32^, the potential where more Nb ions locate is deeper than that of less Nb ions. Thus, an unsymmetrical model of the minima of the Gibbs free energy (*G*) should be applicable in a micro local region with different Nb concentrations. Due to the compositional gradient of Nb ions, it is reasonable to consider a continuous deeper potential along the *y-axis*, delineated as Fig. 3(b). For a local region where the potential energy Δ*G*_1_ < Δ*G*_2_, the polarization is along the *y*-axis, following the direction of Δ*n*. For amount of proximate local regions, where Δ*G*_1_ < Δ*G*_2_ < Δ*G*_3_ < Δ*G*_4_<…, a macroscopic polarization is rational to form in the corresponding direction. And then, a polarization field component ***P***_Δ*n*_ is determined by the continuous gradient potential. Consequently, we illustrate the vector sum ***P*** of ***P***_Δ*T*_ induced by the temperature gradient field and ***P***_Δ*n*_ caused by the refractive index gradient under both conditions of the temperature increasing and decreasing, as depicted in Fig. 3(c) and (d), respectively. Whereas ***P*** orients to neither the *x*-axis nor the *y*-axis, lasers at both TM and TE polarization can respond to be depolarized. In Fig. 2, it should be noted that at temperatures a bit lower than T^*^, the correlation between nanostructures is rather weak, which means that the depolarization of incident beams is not obvious. At lower temperatures around *T*_m1_ and *T*_m2_, due to strengthened correlation, depolarization becomes more notable.

**Figure 4:**
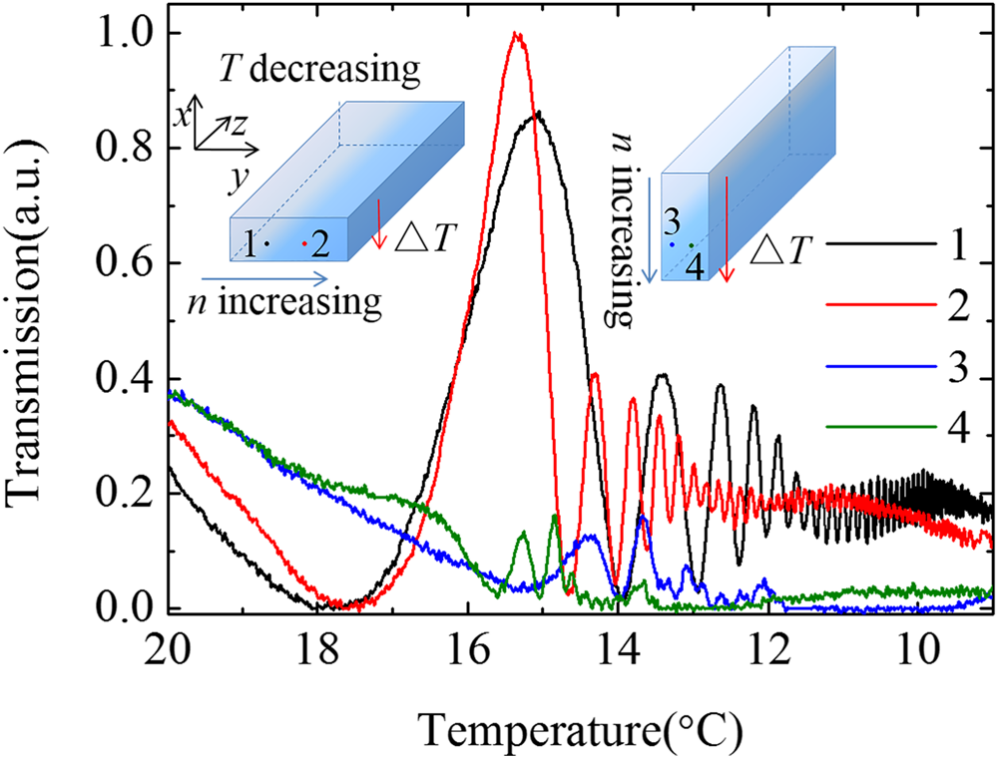
Polarization modulation under different placements of the crystal with utilizing a 5-mW laser at TM polarization.

To further confirm the impact of ***P***_Δ*n*_ on the experimental results, the comparison of the transmitted light intensity is made between two different placements of the crystal as shown in the insets of Fig. 4. The incident points are chosen in the horizontal direction for each placement, named as 1, 2, 3 and 4, respectively. The incident beam used is TM-polarized with the crystal temperature controlled decreasingly. For lasers with the same incident power (~5 mW), the performances are significantly different for these two setups. For the case of points 1 and 2, which are located along the direction of Δ*n*, it shows obvious depolarization consistent with the results displayed in Fig. 2. Nevertheless, for the case of points 3 and 4, for which the locations are orthogonal to the Δ*n* direction, the ***P***_Δ*n*_ and ***P***_Δ*T*_ are both in the vertical direction. Thus, the depolarization is rather weaker compared with that of points 1 and 2. For the case of points 3 and 4, the weak peaks should be caused by little deviation of the direction of Δ*n* from the vertical direction.

### Quasiperiodic intensity oscillation induced by the temperature-dependent refractive index

Theoretically, a quasiperiodic changing of the direction of ***P*** in Fig. 3 should be inferred to result in a regular oscillation. Nevertheless, as have been discussed above, ***P***_Δ*T*_ and ***P***_Δ*n*_ are both in association with the correlated disorder state. With the temperature decreasing, the correlation between the nanostructures will strengthen, making both ***P***_Δ*T*_ and ***P***_Δ*n*_ lager in value. Thus, it is inconceivable to consider a quasiperiodic changing direction of ***P***. Hence, we focus to another possible factor, namely the temperature-dependent concentration of the PNDs, which is responsible for the polarization modulation in the previous report regarding the EO effect of KTN crystals^23^.

**Figure 5:**
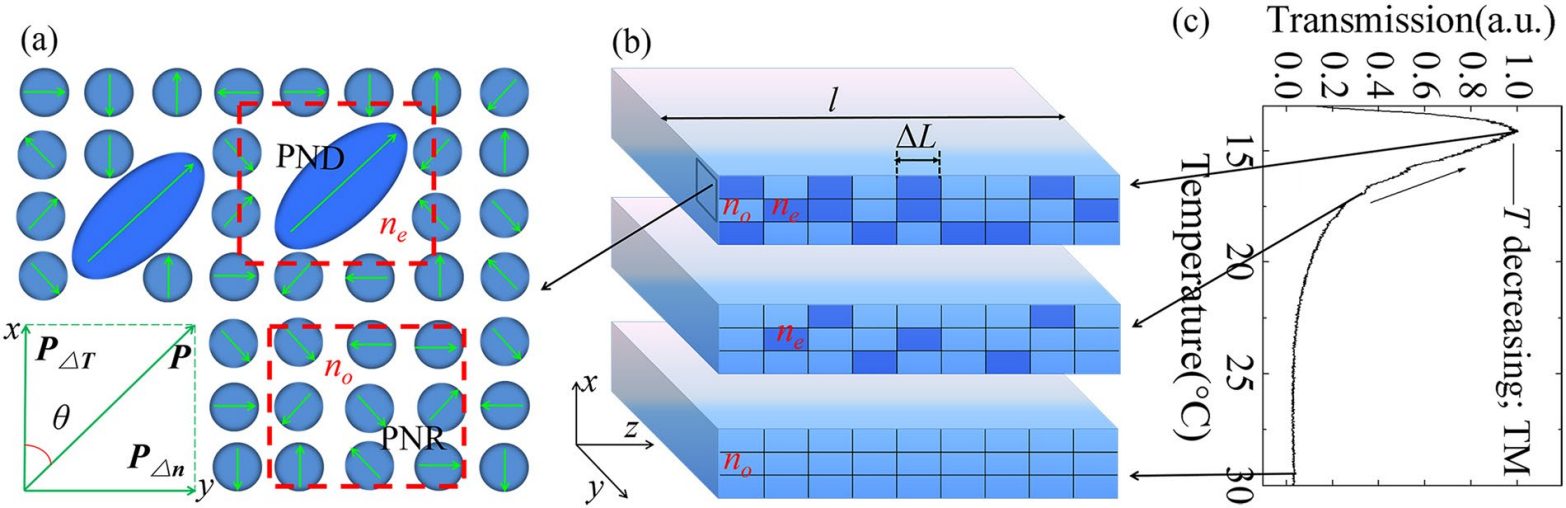
(**a**) Diagram of correlated disorder nanostructures of the crystal and the polarization fields induced by the temperature gradient and the refractive index gradient in the range of [*T*_f_, T^*^]. (**b**) Divided regions with different refractive indexes, *n*_e_ and *n*_o_. (**c**) Transmission change with the temperature decreasing from 30 °C to 13 °C.

For simplicity, the direction of ***P*** is taken as a permanent *θ* angle to ***P***_Δ*T*_, as shown in Fig. 5(a). It describes locally the polarization of the nanostructures under correlated disorder state in *x*-*y* plane. In the range of [*T*_f_, *T* ^*^], some PNRs will combine into the PNDs. The PNRs fluctuate randomly activated by thermal motion, presenting zero polarization in time and space, while the PNDs exhibit none zero polarization in long time scale^9^. When an induced field ***P*** is involved, the PNDs will respond to align with it, but the PNRs stay in random fluctuation due to thermal activation. Thus, regions constituted by the PNDs will show anisotropy along ***P*** and its normal direction, with different refractive indexes for certain polarization components. In Fig. 5(b), the sample is divided along *z-*axis into *N* local regions in length of Δ*L* with different refractive indexes (i.e., *n*_e_ and *n*_o_), representing the regions of PNDs and PNRs, respectively. As the PNDs and the PNRs are both in submicron scale or even smaller, it is logical that *N* = *l*/Δ*L* ≫ 1. In addition, due to their random distribution, we assign *ρ*(*n*_*e*_) and *ρ*(*n*_o_) to the concentration of the PNDs and the PNRs, where *ρ*(*n*_*e*_) + *ρ*(*n*_o_) = 1. At a particular temperature, the optical paths are *n*_e_*Nρ*(*n*_e_)Δ*L* + *n*_o_*N*[1 − *ρ*(*n*_e_)]Δ*L* and *n*_o_*l* for the polarization components parallel and normal to ***P*** respectively, whereupon the phase difference between the two polarizations can be formulated as:

1 $$\begin{array}{rcl}{\rm{\Delta }}\phi  & = & \frac{2\pi }{\lambda }\{{n}_{e}N\rho ({n}_{e}){\rm{\Delta }}L+{n}_{o}N[1-\rho ({n}_{e})]{\rm{\Delta }}L-{n}_{o}l\}\\  & = & \frac{2\pi }{\lambda }({n}_{e}-{n}_{o})\rho ({n}_{e})\end{array}$$

Substitute Eq. (1) to $I={I}_{0}{\sin }^{2}({\rm{\Delta }}\phi /2)$, the well-known formula in wave optics, the transmitted light intensity is obtained as below:

2 $$I={I}_{0}{\sin }^{2}[\frac{\pi }{\lambda }({n}_{e}-{n}_{o})\rho ({n}_{e})]$$

When the temperature decreases, the concentration of the PNDs will increase continuously and saturate at a certain concentration to percolate to the ferroelectric domains at *T*_f_^33^. In addition, as the PNDs scatter light obviously^34^, the transmitted light intensity is negatively correlated to *ρ*(*n*_*e*_). Assume the saturation concentration as *ρ*_*f*_(*n*_*e*_), the transmitted intensity can be multiplied by an factor $1-\rho ({n}_{e})/{\rho }_{f}({n}_{e})$, then we obtain

3 $$I={I}_{0}[1-\frac{\rho ({n}_{e})}{{\rho }_{f}({n}_{e})}]{\sin }^{2}[\frac{\pi }{\lambda }({n}_{e}-{n}_{o})\rho ({n}_{e})]$$

Since *ρ*(*n*_*e*_) and *T* are negatively correlated in the range of [*T*_f_, T^*^], the intensity oscillation can be explained accordingly. At T^*^ = 30 °C, there only exists PNRs rather than PNDs, which means that *ρ*(*n*_*e*_) and *I* are both equal to 0, and the crystal shows no anisotropy. At a lower temperature such as 17 °C, with more PNDs emerging and *ρ*(*n*_e_) increasing, Δ*φ* ceases to be 0 and *I* increases. When the temperature keeps lowering to 14.2 °C, Δ*φ* increases to its first integer multiple of *π*/2 with further increment of the PNDs concentration, and the transmitted intensity reaches its first peak value. The explanation is schematically delineated in Fig. 5(b) and (c). For the peaks getting increasingly weaker during the oscillation at lower temperatures, it can be attributed to the more and more serious scattering when *ρ*(*n*_*e*_) increases.

### Repeatability of the experiment

**Figure 6:**
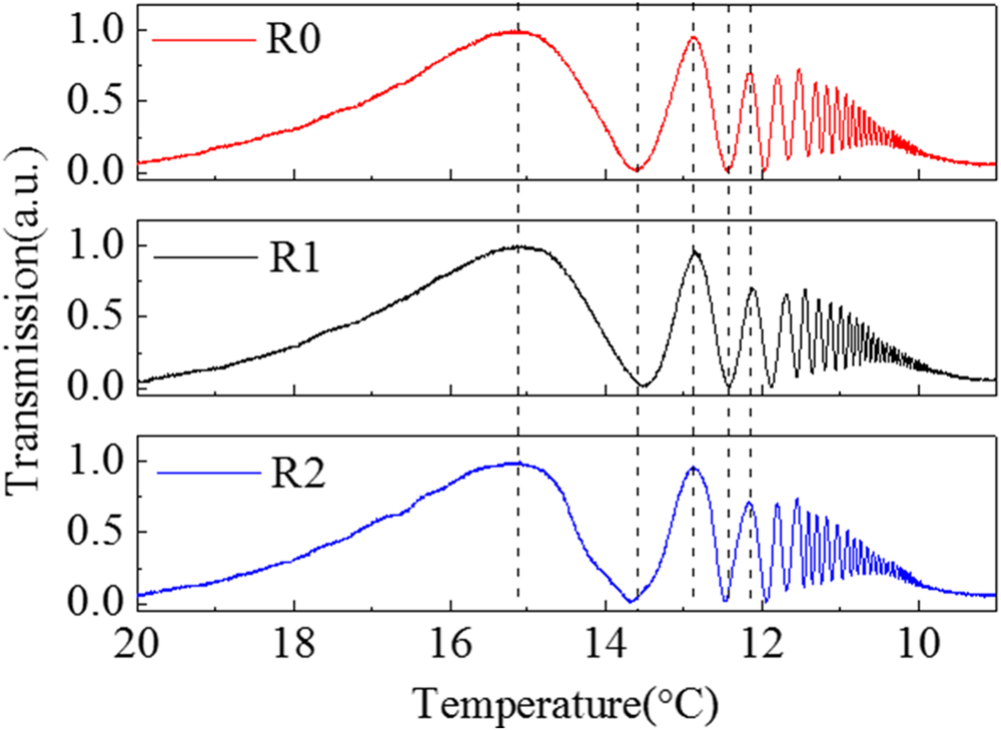
Repeated experiments regarding the abnormal optical anisotropy, with a 3 °C/min decreasing rate from 20 °C to 9 °C with TM-polarized lasers (the red, the black and the blue curves respectively represent results at the same experimental conditions).

Considering that the correlated disorder state of the crystal involves a random process, it is necessary to double-confirm the experimental consistency. Therefore, the temperature-dependent experiment is repeated for several times when the temperature decreases from 20 °C to 9 °C, as presented in Fig. 6. With comparison of the three curves, the peaks and troughs can be observed to correspond perfectly, which is a solid evidence that the anisotropy of the crystal is determined at certain temperature, despite that the correlated disorder presents a random characteristic. This is crucial for both practical use and theoretical research.

## Conclusion

We report an abnormal optical anisotropy in Cu:KTN crystals. The origin of this unique phenomenon is attributed to the cooperation between the temperature gradient field and the refractive index gradient field. Microscopically, it is a result of the correlated disorder characteristic of KTN crystals in the range of [*T*_f_, T^*^]. Since the refractive index gradient is responsible for ***P***_**△***n*_, the tunable ***P***_**△***n*_ is accessible by a tuned refractive index gradient. This will lead to a controllable optical anisotropy in KTN crystals or other relaxors, which is meaningful to the development of a wide range of functional devices. In addition, the mechanism reported in this paper will be also beneficial for completing the correlated disorder theory of ferroelectric relaxors.

## Methods

### Preparation of the sample and dielectric constant measurement

The Cu:KTN crystals used in the experiment have been achieved through the top-seeded solution growth method^35^, with 0.1% mole Cu-ions doped. The doping of Cu ions will enables the single crystal owning higher dielectric property than pure KTN with decreasing the resistance of the grain^36^. It is cut into a cuboid shape in the size of 1.89(*h*) × 3.38(*w*) × 5.73(*l*) mm^3^ along the basic crystallographic directions with faces optical-polished. In the measurement of the *T*-dependent *ε*_r_, the two faces *(w* × *l)* are coated with silver electrodes. Then, it is connected to an LCR meter with a 1 kHz, 1 V sinusoidal signal. The temperature of the crystal is set by a Peltier which is linked to a temperature controller (TEC Source 5300) with 0.01 °C precision.

### The cross-polarization setup used in the experiment

**Figure 7:**
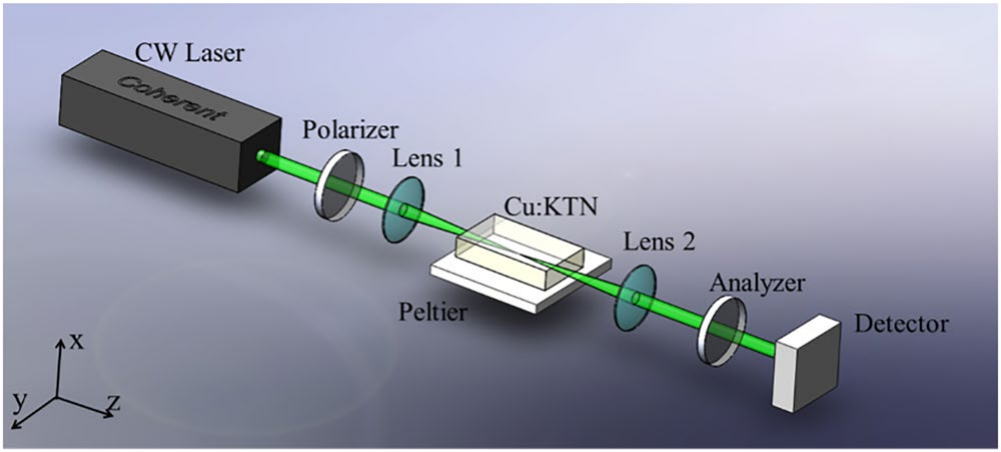
Diagram of the experimental setup regarding the abnormal optical anisotropy.

As shown in Fig. 7, a polarized 532 nm laser is focused into the Cu:KTN sample. Then, it is defocused by a lens and traverses an analyzer. A photodetector is set to probe the transmitted intensity signals. During the testing process, the crystal is positioned with its geometrical axes according to the basic crystallographic directions. The light beam is normally incident to the *x-y* face. The polarizer is along with either the *x* or the *y* direction, instead of a 45° cross-polarization setup used in routine studies of optical anisotropy.
